# Independent association of thigh muscle fat density with vascular events in Korean adults

**DOI:** 10.1186/s12933-024-02138-w

**Published:** 2024-01-28

**Authors:** Hun Jee Choe, Won Chang, Matthias Blüher, Steven B. Heymsfield, Soo Lim

**Affiliations:** 1 Department of Internal Medicine, Hallym University Dongtan Sacred Heart Hospital, Hwaseong, South Korea; 2 Seoul National University College of Medicine, Seoul, South Korea; 3 Department of Radiology, Seoul National University Bundang Hospital, Seongnam, South Korea; 4 Helmholtz Institute for Metabolic, Obesity and Vascular Research of the Helmholtz Zentrum München at the University of Leipzig and University Hospital, Leipzig, Germany; 5 Pennington Biomedical Research Center, Baton Rouge, LA USA; 6 Department of Internal Medicine, Seoul National University College of Medicine, Seoul National University Bundang Hospital, 82, Gumi-ro, 173 Beon-gil, Bundang-gu, Seongnam, 13620 South Korea

**Keywords:** Myosteatosis, Ectopic fat, Coronary artery disease, Cardiovascular disease

## Abstract

**Background:**

We aimed to explore the associations between thigh muscle fat density and vascular events.

**Methods:**

A total of 3,595 adults (mean age, 57.2 years; women, 1,715 [47.7%]) without baseline cardiovascular events from the Korean Atherosclerosis Study-2 were included. Muscle and fat area at the mid-thigh level were measured by computed tomography (CT) using the following Hounsfield Unit range: 0–30 for low density muscle (LDM); 31–100 for normal density muscle (NDM); and − 250 to − 50 for fat.

**Results:**

During a median follow-up period of 11.8 (4.3–13.9) years, vascular events occurred in 11.6% of men and 5.9% of women. Individuals with vascular events had a larger LDM area (men: 48.8 ± 15.5 cm^2^ vs. 44.6 ± 14.5 cm^2^; women: 39.4 ± 13.2 cm^2^ vs. 35.0 ± 11.8 cm^2^, both *P* < 0.001) compared with those who did not have vascular events during the follow-up of at least 5 years. The LDM/NDM ratio was also independently associated with vascular events after adjusting for cardiometabolic risk factors. Moreover, the LDM/NDM ratio improved the prognostic value for vascular events when added to conventional risk factors.

**Conclusions:**

The current study suggests that a higher thigh muscle fat infiltration is associated with an increased risk of developing vascular events among Korean adults.

**Supplementary Information:**

The online version contains supplementary material available at 10.1186/s12933-024-02138-w.

## Introduction

Several recent studies report that estimates of visceral adipose tissue (VAT) have larger magnitude correlations with clinical risks of obesity than do commonly used biometrics such as body mass index (BMI) [1, 2]. The purported mechanism underlying this observation is that VAT and related intrahepatic and intramuscular fat have systemic actions and thus differ from ectopic fat located nearby and around the heart, blood vessels, and kidney that are thought to act locally within the adjacent organs [3]. Alternatively, it is suggested that intraorgan and periorgan fat reflect an impaired adipogenesis state during positive caloric balance [4]. This impairment triggers adverse endocrine and immune processes, contributing to metabolic disease and atherosclerosis [5].

Skeletal muscle occupies a large portion of body mass and is the primary site for insulin-stimulated glucose uptake [6]. Previous studies on the relationship between intramuscular fat and metabolic risk factors had several limitations as only a small number of participants with specified disorders were included in the samples [7–9]. There have also been studies on the relationship between the accumulation of intramuscular fat and insulin resistance that examined these associations using the contemporary technologies including nuclear magnetic resonance spectroscopy, immunofluorescence microscopy, and histochemical examination with biopsied specimens [10, 11]. The results of these studies were inconsistent, however, and analyses were not corrected for BMI or whole body fatness.

In the Framingham Heart Study that included 2,945 participants (mean age, 50.8 years, 50.2% women), computed tomography (CT) attenuation values in the paraspinous muscles were associated with hyperglycemia, dyslipidemia, and hypertension in both men and women in a multivariate model that was adjusted for BMI and VAT [12]. Similarly, a study involving 75 Asian participants with obesity (mean age, 41.9 years; 69.3% women) reported a significant correlation between insulin resistance and low density muscle (LDM) area at the mid-thigh level. Importantly, this association remained significant even after controlling for markers of adiposity [7]. To date, few studies have explored the potential link between LDM at the mid-thigh level and the incidence of vascular events in a large population sample. The aim of this study was to investigate the relationship between fat deposits in the thigh muscles, cardiometabolic risk factors, and vascular events.

## Methods

### Demographics and anthropometric measurements

The Korean Atherosclerosis Study is an ongoing longitudinal cohort with the baseline survey and examination conducted in people who underwent routine health checkups at Seoul National University Bundang Hospital (SNUBH), Seongnam, Korea in 2006–2014 [13]. Of the 3,749 individuals with one or more cardiometabolic risk factors, 140 patients with previous vascular events and 14 patients without CT scan at the LDM level were excluded from the analysis. The cardiac CT scans, recommended for participants with cardiovascular risk factors, were carried out between 2006 and 2014 primarily for research purposes. During this period, an additional single cut from the abdomen and mid-thigh level was obtained with the consent of the participants enrolled in the study. The incidence of vascular event was screened from 2006 to 2021. This study was conducted in accordance with the ethical standards of the Declaration of Helsinki. The study protocol was approved by the Institutional Review Board of Seoul National University Bundang Hospital (B-0809-061-103). All participants agreed to participate in the study and provided written consent.

At baseline examination, self-completed questionnaires were used to assess the participants’ demographic, general health, and social history (e.g., alcohol consumption, smoking status, frequency of exercise, etc.). Height and body weight were measured on an electronic scale with the participants wearing light indoor clothing. BMI was calculated as weight (kg) divided by height (m) squared. Systolic and diastolic blood pressures were measured with an electronic blood pressure monitor (UA-1020 device; A&D, Tokyo, Japan) while the participants were seated. Blood pressure was measured twice at a 5-minute interval, and the mean value was used in the analysis.

### Definition of disease status

The definition of vascular event included clinical events such as coronary artery disease (CAD), cerebrovascular disease (CeVD), peripheral artery disease (PAD), and significant stenosis of coronary arteries by imaging modalities. CAD was defined as development of acute coronary syndrome (unstable angina and myocardial infarction) or if coronary artery stenosis (> 50%) was detected on the cardiac CT. The diagnosis of CeVD included clinical diagnosis of ischemic stroke or significant narrowing of cerebral arteries in brain imaging. PAD was defined by revascularization or thrombolysis in peripheral arteries, or the presence of significant narrowing or obstruction or ulcerated plaque at or below the internal iliac arteries by imaging.

A BMI of 25 kg/m^2^ or higher was defined as obesity based on the WHO guidelines for the Asia-Pacific region [14]. Visceral obesity was defined as VAT area ≥100 cm^2^ in the abdominal CT scan. Diagnosis of dyslipidemia was made upon use of lipid-lowering agents (statin, fibrate, niacin, ezetimibe, or omega-3 fatty acid), or diagnosis of hyperlipidemia made by a physician. Diabetes mellitus was assessed based on the laboratory values of fasting plasma glucose ≥126 mg/dL, HbA1c ≥6.5%, or 2-hour postprandial glucose ≥200 mg/dL, any current use of antidiabetic medication, or previous diagnosis by a physician. Hypertension was defined if the blood pressure was 140/90 mmHg or higher, or if the participant was taking antihypertensive medications.

### Cardiac multidetector CT

Baseline cardiac CT was performed with a 64–detector row CT scanner (Brilliance 64; Philips Medical Systems, Best, the Netherlands) as previously described [15]. The tube voltage was 100 kVp, and slice thickness 5 mm with a 5 mm slice increment. In participants with a heart rate of > 70/min, heart rate was reduced using 10–30 mg of intravenous esmolol (Brevibloc; Jeil Pharmaceutical, Seoul, Korea). Images in the mid-diastolic phase of the cardiac cycle were reconstructed, and coronary arteries larger than 1.5 mm in diameter was evaluated for degree of stenosis, plaque severity, and characteristics in all three coronary vessels. A follow-up cardiac CT was conducted at the physician’s discretion when patients had chest discomfort or exhibited a high cardiovascular risk.

### Abdominal muscle and adipose tissue area measurement

Abdominal VAT and subcutaneous adipose tissue (SAT) areas were quantified using standard protocols at the umbilical level on non-contrast abdominal scan slices obtained during the cardiac CT scan [16] with the following parameters: kVp: 100; mAs: 95; dose right index: 13; pitch: 0.674; rotation time: 0.5 s; dose right automatic current setting (ACS). Using dedicated software (Radipia; 3DMED, Seoul, South Korea), HU cutoff values of − 250 to − 50 were assigned for total adipose tissue on the CT images. Abdominal SAT area was calculated by subtracting the area of VAT from the total adipose tissue (TAT) area.

### Mid-thigh muscle and adipose tissue area measurement

**Fig. 1:**
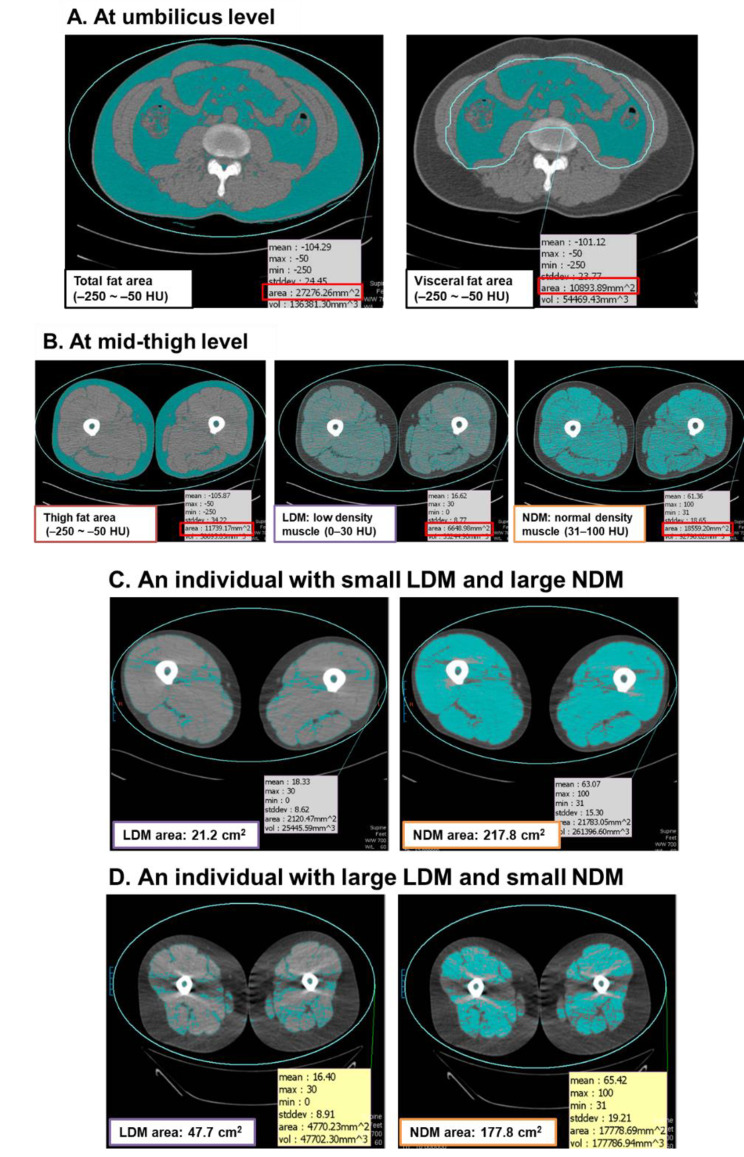
**(A)** Measurement of visceral fat area at umbilical level. **(B)** Measurement of fat area and low density muscle (LDM) and normal density muscle (NDM) area at mid-thigh level. C and D. Representative photos of individuals who had small LDM and large NDM area **(C)** and who had large LDM and small NDM area **(D)**

Images of the mid-thigh level were additionally obtained for each individual from a single noncontrast cross-sectional scan acquired during cardiac CT (Fig. 1) using the following parameters: kVp: 100; mAs: 95; dose right index: 13; pitch: 0.685; rotation time: 0.5 s; dose right ACS. The mid-thigh level was precisely determined as the midpoint between the anterior superior iliac spine and the knee joint. Similarly, pixels between − 250 HU and − 50 HU were labeled as thigh adipose tissue. Skeletal muscle attenuation at the thigh level was determined by measuring the mean attenuation value within the range of 0–100 HU. Images were further compartmentalized according to the distribution of attenuation values representing low and normal muscle density. LDM was defined as the cross-sectional area of mid-thigh tissue with a HU of 0–30, and normal density muscle (NDM) was defined as 31–100 HU, corresponding to the muscle pixels with attenuation values within two standard deviations (SDs) of the mean attenuation value observed in healthy, lean muscle [17]. Mid-thigh LDM/NDM ratio was calculated by dividing the area of LDM by corresponding NDM area at the same level.

The intraobserver and interobserver variabilities were assessed using the intraclass correlation coefficient and Bland–Altman plots. No significant variabilities were found in the measurement of LDM and NDM (Additional file 1: Table S1, Figure S1).

### Biochemical measurements

Baseline blood samples were drawn after at least 10 h of fasting, and the collected samples were centrifuged at 1006 *g* for 10 min at 4 °C. Plasma glucose level was analyzed by the hexokinase method, while high-density lipoprotein (HDL)-cholesterol and low-density lipoprotein (LDL)-cholesterol levels were measured by homogeneous enzymatic assays, and triglyceride level was measured by a glycerol-3-phosphate oxidase peroxide method. An XE-2100 Hematology Analyzer (Sysmex Corp., Kobe, Japan) was used to measure white blood cell count, hemoglobin level, hematocrit level, and platelet count. Aspartate and alanine aminotransferase (NADH-UV method), total bilirubin (bilirubin oxidase method), blood urea nitrogen (urease/glutamate dehydrogenase method), creatinine (Jaffe’s kinetic method) levels were measured at the central laboratory of SNUBH. The biochemical tests were performed immediately after sample collection. Plasma insulin and C-peptide were measured by radioimmunoassay (Linco St. Louis, MO, USA), and HbA1c was measured using a HPLC method as reported previously (Bio-Rad, Hercules, CA, USA) [18].

### Statistical analysis

Data are presented as mean ± SD for continuous variables, or numbers and percentages for categorical variables. Differences in means and proportions by vascular event status were tested using Student’s *t* tests or Chi square tests. For continuous variables that do not follow normal distributions, data were log transformed to achieve normality. All analyses were stratified by sex.

To assess for cardiovascular risk factors, participants were divided into four groups according to sex-specific quartiles for LDM/NDM ratio (%), LDM area (cm^2^), VAT area (cm^2^), and BMI (kg/m^2^), respectively. The Cochran Armitage test was used for trend analyses. Possible associations among various measures were analyzed using Pearson’s correlation.

To stratify participants in the Kaplan–Meier analysis based on the LDM/NDM ratio, an optimal cutoff value was derived using a receiver operating characteristic (ROC) curve analysis. The optimal cutoff point for the LDM/NDM ratio was determined as the point that maximized the product of sensitivity and specificity in the area under the ROC curve (AUC) for men and women, respectively. These cutoff points for the LDM/NDM ratio were applied in the Kaplan–Meier curve to observe vascular events. The log-rank test was used to assess the significance of the differences in event-free survival observed between the two groups.

The risk of developing vascular events was assessed using Cox proportional hazard models. Variance inflation factor (VIF) ≥2 was regarded as indicating multicollinearity. Variables with VIF < 2 were considered acceptable and were used in the univariable and multivariable Cox regression models. Each cardiovascular risk factor was assessed through separate univariable Cox regressions; to describe how the factors jointly impact on the development of vascular events, all risk factors including the LDM/NDM ratio were incorporated into multivariable models.

C statistics were calculated to test the improvement in the discriminative ability of the LDM/NDM ratio, in addition to the established cardiovascular risk factors. AUCs were computed using the cardiovascular risk factors that were significant in the multivariable Cox regression models. The improvement in the change in the AUC afforded by the addition of the LDM/NDM ratio was calculated using DeLong’s test.

Statistical significance was defined as *P* < 0.05. Statistical analyses were performed using SPSS software package (version 28.0; IBM, Armonk, NY, USA) and R v4.0.5 (R Foundation for Statistical Computing, Vienna, Austria).

## Results

### Baseline demographics and anthropometric measurements

The baseline characteristics of the study participants are shown in Table 1 separately for men and women. Mean age of the sample was 57.2 ± 15.9 years and mean BMI was 25.5 ± 4.0 kg/m^2^. At the baseline, 1,569 participants (43.5%) were on lipid-lowering medication, 689 (19.1%) were on diabetes medication, and 536 (14.9%) were on antiplatelet therapy. Changes in medication during the follow-up were not consistently tracked because some patients received medications at local clinics.

**Table 1:** Baseline characteristics of the participants with vascular events or with >5 years of event-free years of follow-up

	Men (*N* = 1,450)			Women (*N* = 1,235)		
	Vascular event (-)	Vascular event (+)	*P*	Vascular event (-)	Vascular event (+)	*P*
	*N* = 1,233	*N* = 217		*N* = 1,132	*N* = 103	
Age (years)	55.1 ± 14.0	61.7 ± 12.5	< 0.001	57.4 ± 13.4	65.5 ± 12.8	< 0.001
BMI (kg/m^2^)	25.5 ± 3.5	25.8 ± 3.6	0.267	25.1 ± 4.1	25.7 ± 4.1	0.148
SBP (mmHg)	130 ± 15	131 ± 16	0.257	129 ± 16	132 ± 16	0.082
DBP (mmHg)	79 ± 11	76 ± 11	0.002	76 ± 11	74 ± 10	0.097
HbA1c (%)	6.6 ± 1.3	7.3 ± 1.6	< 0.001	6.5 ± 1.2	7.4 ± 1.7	< 0.001
Fasting glucose (mg/dL)	123 ± 38	136 ± 49	< 0.001	114 ± 34	132 ± 46	0.001
Total cholesterol (mg/dL)	192 ± 36	180 ± 42	< 0.001	201 ± 39	185 ± 37	< 0.001
Triglyceride (mg/dL)	159 ± 115	152 ± 100	0.678	136 ± 83	150 ± 69	0.028
HDL-cholesterol (mg/dL)	50 ± 12	48 ± 11	0.031	57 ± 14	50 ± 10	< 0.001
LDL-cholesterol (mg/dL)	107 ± 29	99 ± 33	0.009	107 ± 29	96 ± 29	0.002
Total protein (g/dL)	7.2 ± 0.5	7.1 ± 0.5	0.022	7.2 ± 0.4	7.1 ± 0.5	0.021
AST (IU/L)	29 ± 84	31 ± 68	0.773	24 ± 14	24 ± 12	0.652
ALT (IU/L)	36 ± 79	37 ± 89	0.855	26 ± 22	26 ± 17	0.892
Creatinine (mg/dL)	1.11 ± 0.24	1.13 ± 0.22	0.368	0.88 ± 0.19	0.94 ± 0.22	0.028
eGFR (mL/min/1.73 m^2^)	76.4 ± 15.8	73.8 ± 16.3	0.036	74.0 ± 17.1	68.0 ± 16.4	0.002
Urinary microalbumin/Cr	37 ± 160	107 ± 300	< 0.001	31 ± 135	78 ± 201	0.001
hsCRP (mg/L)	0.40 ± 1.47	0.99 ± 2.56	< 0.001	0.21 ± 0.50	0.85 ± 2.01	< 0.001
*Comorbidity*						
Hypertension, n (%)	431 (35.0)	132 (60.8)	< 0.001	413 (36.5)	67 (65.0)	< 0.001
Dyslipidemia, n (%)	523 (42.4)	155 (71.4)	< 0.001	559 (49.4)	76 (73.8)	< 0.001
Diabetes mellitus, n (%)	590 (51.5)	137 (69.9)	< 0.001	431 (41.2)	63 (67.0)	< 0.001
*Social history*						
Regular drinker, n (%)	235 (19.1)	39 (18.0)	0.706	21 (1.8)	0 (0.0)	N/A
Smoking			0.107			0.005
Never smoker, n (%)	741 (60.1)	120 (55.3)		1,107 (97.8)	96 (93.2)	
Ex-smoker, n (%)	299 (24.2)	54 (24.9)		16 (1.4)	4 (3.9)	
Current smoker, n (%)	193 (15.7)	43 (19.8)		9 (0.8)	3 (2.9)	
Exercise, n (%)			0.121			0.280
None	744 (60.4)	142 (65.4)		775 (68.5)	73 (70.9)	
1/week	112 (9.1)	21 (9.7)		48 (4.2)	8 (7.8)	
2–3/week	173 (14.0)	24 (11.1)		122 (10.8)	10 (9.7)	
Daily	203 (16.5)	30 (13.8)		186 (16.4)	12 (11.7)	

BMI, body mass index; SBP, systolic blood pressure; DBP, diastolic blood pressure; HDL, high-density lipoprotein; LDL, low-density lipoprotein; AST, aspartate aminotransferase; ALT, alanine aminotransferase; eGFR, estimated glomerular filtration rate; Cr, creatinine; hsCRP, high sensitivity C-reactive protein; N/A, not applicable.

The participants were followed up every 3–6 months and the median follow-up period was 11.8 (4.3–14.9) years. During the follow-up period, vascular events were detected in 217 (11.5%) of men: 147 (7.8%) had CAD, 56 (3.0%) had CeVD, and 38 (2.0%) had PAD. In women, 103 (6.0%) developed vascular events: 52 (3.0%) had CAD, 36 (2.1%) had CeVD, and 26 (1.5%) had PAD, respectively.

To mitigate the potential bias arising from assuming that the participants who were lost to follow-up were event-free, we conducted a comparison between the individuals who experienced vascular events during the follow-up and those who were followed up for a minimum of 5 years without experiencing vascular events. The men and women who developed vascular events were older and had higher fasting glucose, HbA1c, and hsCRP levels than did those who had no vascular events during at least 5 years of follow-up. The prevalence of albuminuria, hypertension, dyslipidemia, and diabetes mellitus were higher in both men and women who developed vascular events.

Body composition in the abdomen and at the mid-thigh level, as assessed by CT, is provided in Table 2. The abdominal TAT and VAT areas were greater in these individuals than in those without vascular events. Notably, abdominal SAT area was larger in women whereas abdominal VAT area was substantially larger in men.

**Table 2:** Body composition in the abdomen and at the mid-thigh level, as assessed by computed tomography according to vascular event status by sex (participants with vascular events or with >5 years of event-free years of follow-up)

	Men (*N* = 1,450)			Women (*N* = 1,235)		
	Vascular event (-)	Vascular event (+)	*P*	Vascular event (-)	Vascular event (+)	*P*
	*N* = 1,233	*N* = 217		*N* = 1,132	*N* = 103	
**Abdomen**						
Total adipose tissue area (cm^2^)	288.8 ± 110.2	312.1 ± 118.9	0.005	331.2 ± 116.4	367.7 ± 127.4	0.003
Visceral adipose tissue area (cm^2^)	141.5 ± 58.6	154.3 ± 59.6	0.003	113.9 ± 50.7	142.6 ± 56.7	< 0.001
Subcutaneous adipose tissue area (cm^2^)	147.3 ± 69.4	157.7 ± 78.6	0.046	217.3 ± 85.1	225.1 ± 88.3	0.370
**Mid-thigh area**						
Fat area (cm^2^)	90.5 ± 45.2	91.4 ± 42.1	0.781	152.6 ± 63.6	136.1 ± 56.6	0.011
Muscle area (cm^2^)	242.5 ± 34.8	228.8 ± 37.0	< 0.001	172.7 ± 29.1	161.5 ± 33.7	< 0.001
LDM area (0–30 HU) (cm^2^)	44.5 ± 14.3	49.2 ± 15.2	< 0.001	35.3 ± 12.0	39.4 ± 13.2	0.001
NDM area (31–100 HU) (cm^2^)	198.0 ± 32.7	179.7 ± 34.5	< 0.001	137.4 ± 26.4	122.1 ± 31.9	< 0.001
LDM/NDM ratio (%)	23.2 ± 8.7	28.8 ± 11.7	< 0.001	26.7 ± 11.1	35.9 ± 20.0	< 0.001

LDM, low density muscle; NDM, normal density muscle.

**Fig. 2:**
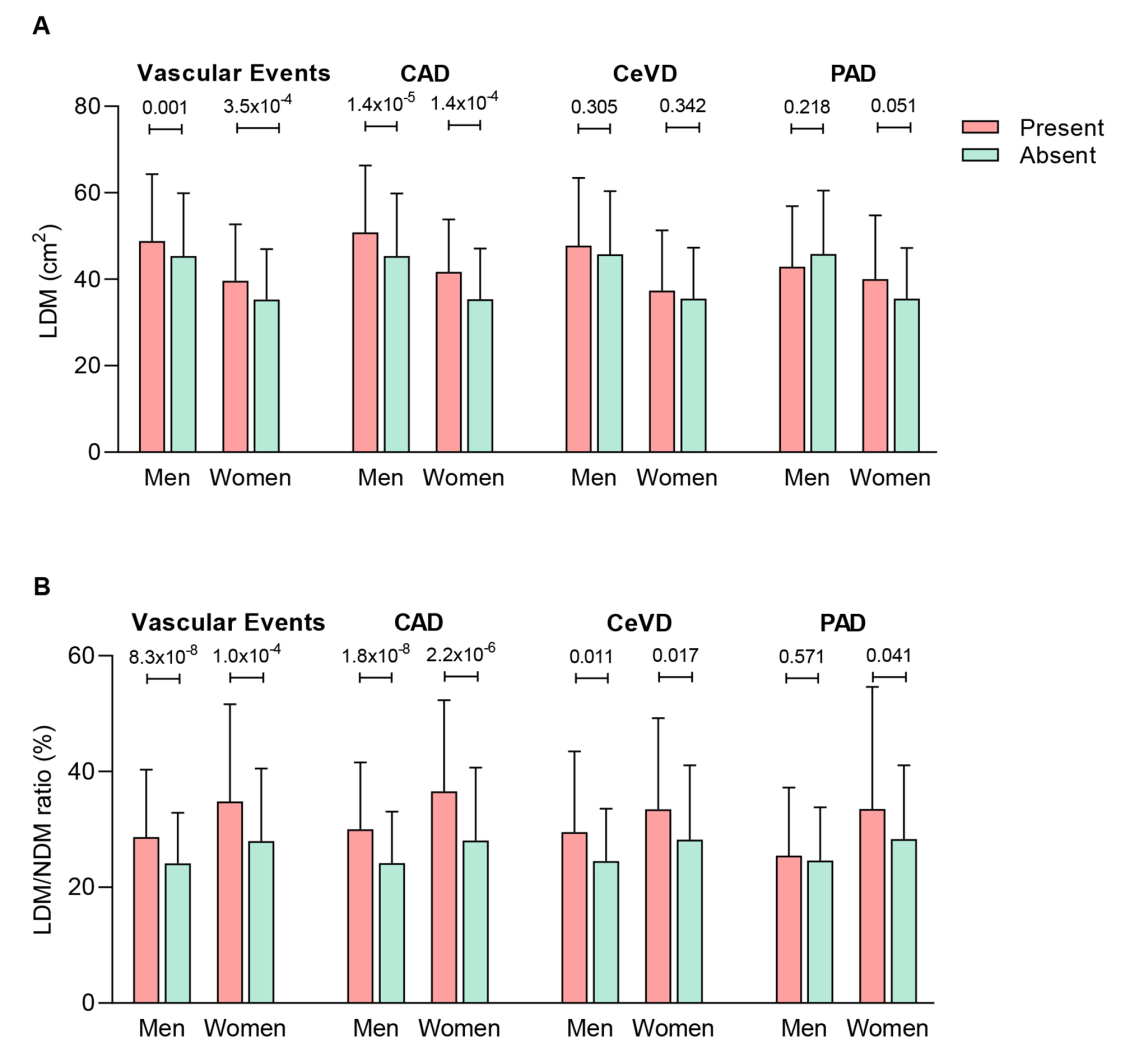
Incidence of vascular event according to **(A)** LDM area (cm^2^) or **(B)** LDM/NDM ratio (%). Red and blue indicates the participants who did or did not develop the disease, respectively. VE, vascular event; CAD, coronary artery disease; CeVD, cerebrovascular disease; PAD, peripheral artery disease. *P* value between the groups is listed above the bars

The muscle area at the mid-thigh level was significantly larger in men vs. women, and the fat area at this level was larger in women vs. men (Table 2). In both men and women, the muscle area at mid-thigh level was significantly lower in people who developed vascular events than those who did not (Fig. 2). Of note, participants who developed vascular events had larger LDM area by 4.2 cm^2^ in men and 4.4 cm^2^ in women (both *P* < 0.001) and lower NDM area by 21.8 cm^2^ in men and 17.1 cm^2^ in women (both *P* < 0.001), compared to those who did not, making the LDM/NDM ratio significantly different by 5.8% in men and 9.7% in women between those with and without vascular events. The LDM area decreased with age in men (*P* for trend = 0.018) but not in women (*P* for trend = 0.094) (Additional file 1: Figure S2).

The baseline characteristics of the 140 participants who were excluded from the analysis because of previous vascular events are provided in Additional file 1: Table S2.

### Association of the LDM/NDM ratio with cardiovascular events

**Fig. 3:**
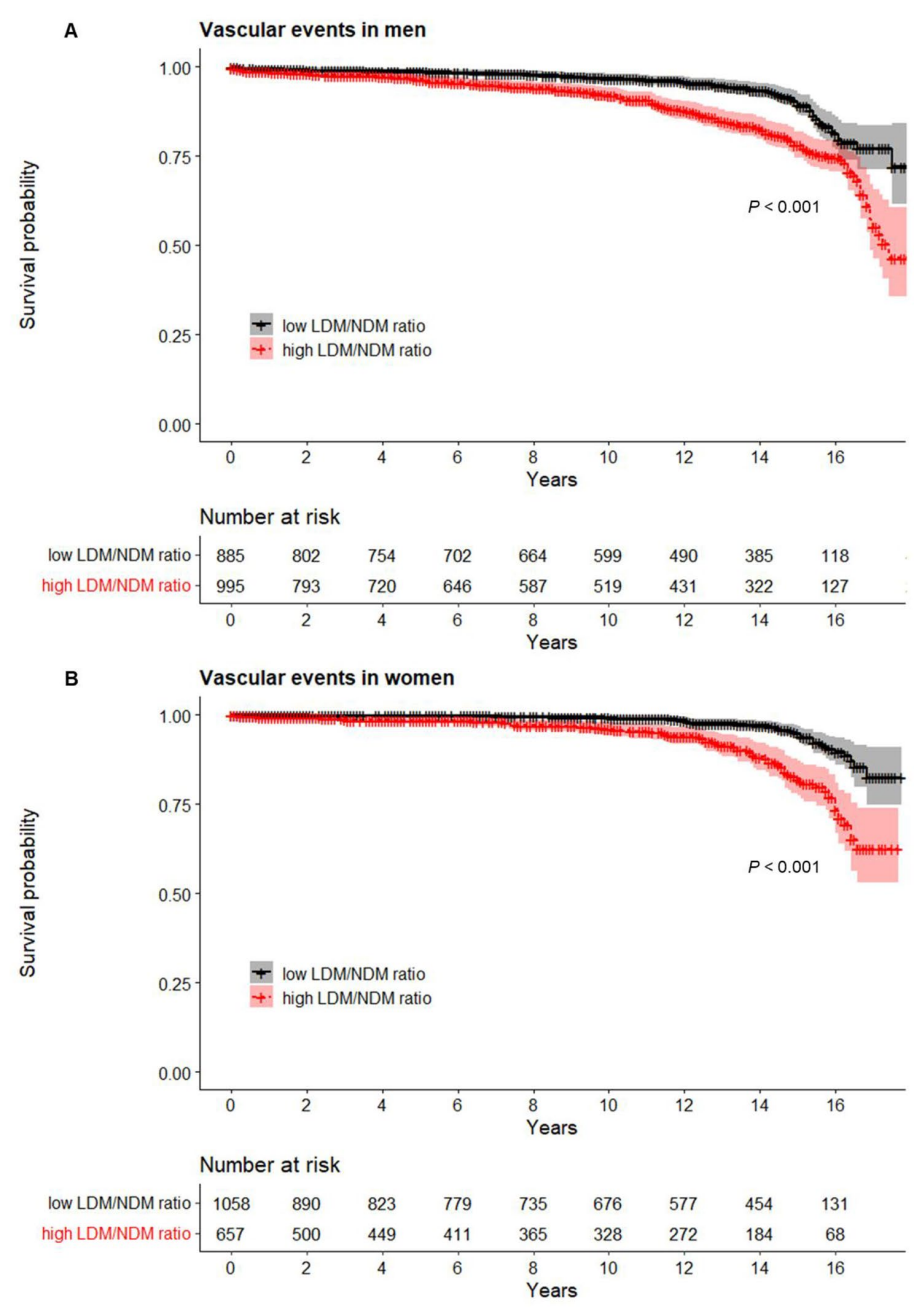
The Kaplan–Meier survival curve demonstrating the incidence of vascular events over the follow-up period, stratified by the optimal cutoff values for LDM/NDM ratio. Participants with high LDM/NDM ratios are represented by black line and low LDM/NDM ratios are in red line, with the 95% confidence interval in shades

The cutoff values for the LDM/NDM ratio derived from the ROC curves were determined to be 22.8% for men and 30.1% for women. Among men, 995 (52.7%) participants exhibited a high LDM/NDM ratio at the baseline, whereas 657 (38.2%) women had a high LDM/NDM ratio at the baseline. Using these established cutoff values, Kaplan–Meier curves were generated to examine the incidence of vascular events over time. Individuals with higher LDM/NDM ratio cutoff values exhibited a significantly elevated incidence of vascular events compared with their counterparts with lower LDM/NDM ratios (*P* < 0.001 for both men and women; Fig. 3).

We evaluated the incidence of vascular events (%) according to the sex-specific quartiles of the LDM/NDM ratio and LDM area. We detected dose-dependent relationships between the quartiles of the LDM/NDM ratio and LDM area and the incidence of vascular events in both sexes (Additional file 1: Figure S3). In particular, a higher proportion of participants with abdominal visceral obesity were diagnosed with vascular events (*P* = 0.002 and *P* < 0.001 for men and women, respectively). However, no increasing trend in vascular events were discovered with higher BMI quartiles (*P* for trend = 0.185, *P* for trend = 0.117 for men and women, respectively). Notably, there was no significant correlation between the LDM/NDM ratio and established cardiovascular risk factors for vascular events in both men and women (Additional file 1: Figure S4).

### Contribution of the LDM/NDM ratio in the development of cardiovascular events

We assessed the significance of the LDM/NDM ratio in the development of vascular events considering established risk factors (Table 3). In the univariate analysis, a higher LDM/NDM ratio, advanced age, an elevated HbA1c level, increased hsCRP levels, a larger VAT area, and the presence of hypertension, dyslipidemia, and chronic kidney disease were significantly associated with vascular events in both men and women. Moreover, lower eGFR was identified as a risk factor in women.

**Table 3:** Risk factors in the development of vascular events

	Men (*N* = 1,880)				Women (*N* = 1,715)			
	Univariable		Multivariable		Univariable		Multivariable	
	Unadjusted	*P*	Adjusted HR	*P*	Unadjusted	*P*	Adjusted HR	*P*
	HR (95% CI)		(95% CI)		HR (95% CI)		(95% CI)	
LDM/NDM ratio (%)	1.06 (1.04–1.07)	< 0.001	1.04 (1.02–1.06)	< 0.001	1.04 (1.03–1.05)	< 0.001	1.02 (1.01–1.04)	0.001
Age (years)	1.04 (1.03–1.06)	< 0.001	1.06 (1.04–1.08)	< 0.001	1.07 (1.05–1.09)	< 0.001	1.08 (1.05–1.11)	< 0.001
HbA1c (%)	1.32 (1.22–1.43)	< 0.001	1.29 (1.14–1.45)	< 0.001	1.36 (1.23–1.50)	< 0.001	1.43 (1.25–1.64)	< 0.001
eGFR (mL/min/1.73 m²)	0.99 (0.98–1.00)	0.195	1.01 (1.00–1.02)	0.155	0.98 (0.97–1.00)	0.019	1.00 (0.99–1.02)	0.788
hsCRP (mg/L)*	1.26 (1.17–1.36)	< 0.001	1.15 (1.06–1.25)	0.001	1.30 (1.17–1.45)	< 0.001	1.09 (0.96–1.24)	0.163
Visceral adipose tissue (cm^2^)	1.00 (1.00–1.00)	< 0.001	1.00 (1.00–1.00)	0.576	1.00 (1.00–1.00)	< 0.001	1.00 (1.00–1.01)	0.848
Hypertension, yes	2.56 (1.95–3.36)	< 0.001	1.45 (1.02–2.07)	0.039	2.61 (1.74–3.92)	< 0.001	1.38 (0.87–2.21)	0.174
Dyslipidemia, yes	2.87 (2.14–3.86)	< 0.001	2.69 (1.83–3.94)	< 0.001	2.31 (1.49–3.59)	< 0.001	2.16 (1.25–3.73)	0.006
Smoking		0.065		0.064		0.034		0.032
Never smoker	1 (Reference)		1 (Reference)		1 (Reference)		1 (Reference)	
Ex-smoker	1.07 (0.77–1.48)	0.686	1.26 (0.85–1.86)	0.243	2.00 (0.73–5.43)	0.176	3.13 (1.02–9.62)	0.046
Current smoker	1.42 (1.01–2.01)	0.047	1.54 (0.96–2.47)	0.077	2.70 (0.85–8.56)	0.091	3.05 (0.41–22.8)	0.277

CI, confidence interval; HR, hazard ratio; LDM, low density muscle; NDM, normal density muscle; hsCRP, high sensitivity C-reactive protein; eGFR, estimated glomerular filtration rate. *Log transformed for analysis

After adjusting for multiple variables concurrently, the LDM/NDM ratio persisted as a significant risk factor for vascular events in both sexes. Age, HbA1c, hsCRP, hypertension, and dyslipidemia also remained independent risk factors in men. Among women, the fully adjusted model indicated that the LDM/NDM ratio, age, HbA1c, hsCRP, dyslipidemia, and smoking significantly contributed to the development of vascular events.

Similar patterns emerged in the univariable analysis of CAD (Additional file 1: Tables S3–S5). The significance of the LDM/NDM ratio was retained in the fully adjusted model. In men, other significant risk factors for CAD included age, HbA1c, hsCRP, and dyslipidemia. In women with CAD, age, HbA1c, hsCRP, dyslipidemia, and smoking were additional significant risk factors.

**Fig. 4:**
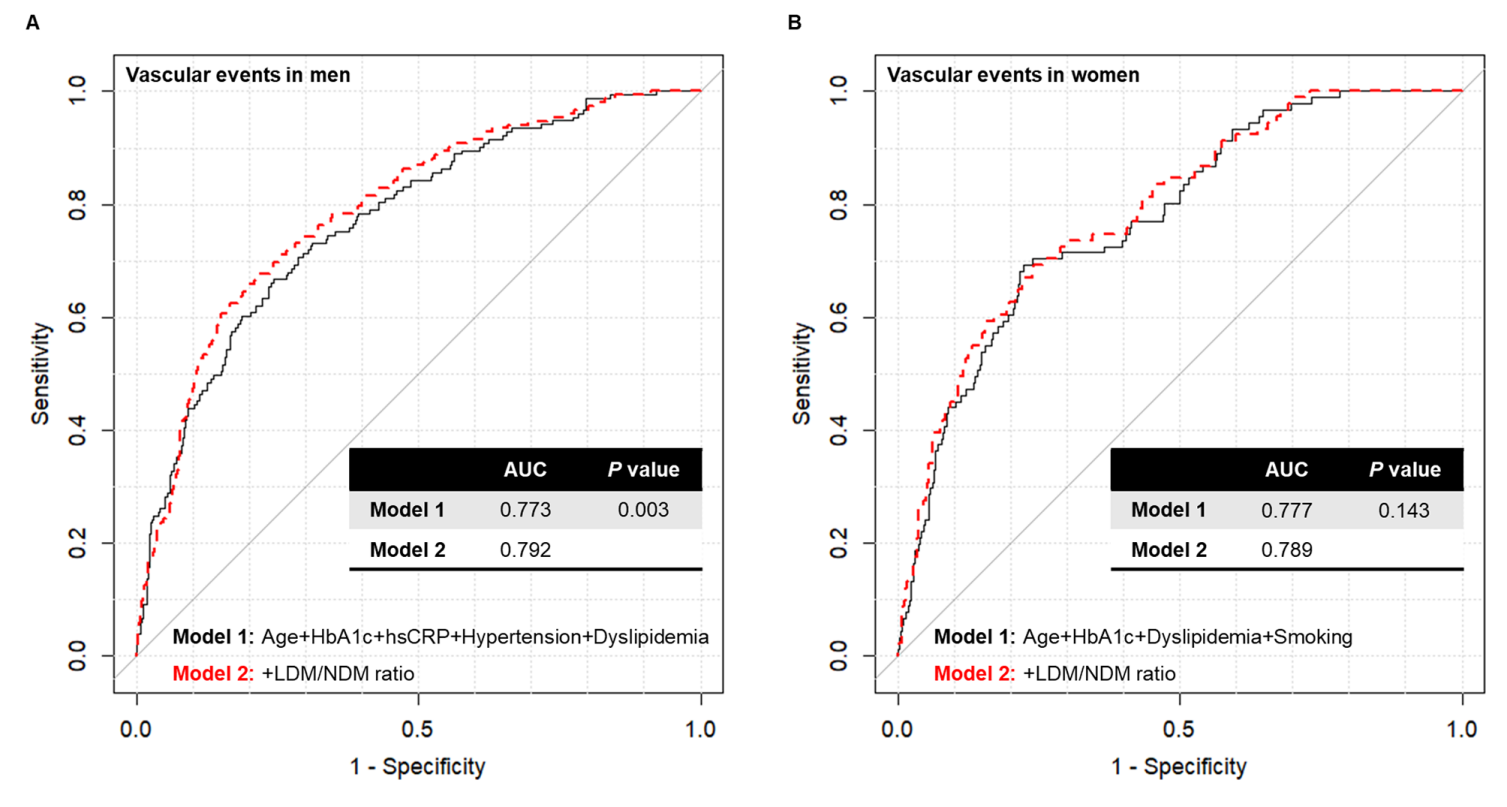
Summary receiver operating characteristic (ROC) curves for predicting vascular events. **(A)** Vascular events in men. Model 1 (black solid line) was adjusted for age, HbA1c, hsCRP, hypertension, and dyslipidemia. Model 2 (red dashed line) was further adjusted for LDM/NDM ratio. **(B)** Vascular events in women. Model 1 was adjusted for age, HbA1c, dyslipidemia, and smoking. Model 2 was further adjusted for LDM/NDM ratio

Next, we evaluated whether the LDM/NDM ratio could effectively improve the prognostic value for the development of vascular events when added to other established cardiovascular risk factors (Fig. 4). In men, the AUC of the ROC for predicting vascular events was 0.773 in the model that integrated age, HbA1c, hsCRP, and the presence of hypertension and dyslipidemia. The addition of the abdominal VAT area to these factors did not improve the predictability of vascular events (Additional file 1: Figure S5). However, after the LDM/NDM ratio was added, the AUC of vascular events in men improved significantly to 0.792 (*P* = 0.003). In women, the AUC of the ROC was modestly improved from 0.777 to 0.789 (*P* = 0.143). The predictability for CAD was significantly improved in both men (*P* = 0.002) and women (*P* = 0.012) after the addition of the LDM/NDM ratio (Additional file S1: Figures S6–S8).

## Discussion

In this study, we found that mid-thigh LDM/NDM ratio, or the cross-sectional area proportion of lipid-infiltrated muscle over healthy muscle of normal density, was significantly greater in persons who developed vascular events. Those with an elevated LDM/NDM ratio exhibited a notably higher rate of vascular events compared to those with a lower ratio, a trend observed in both men and women. Furthermore, the LDM/NDM ratio emerged as an independent prognostic factor for vascular events after adjusting for established cardiovascular risk factors in both genders. Notably, the inclusion of the LDM/NDM ratio alongside conventional risk factors enhanced the overall predictive accuracy of vascular events in men, as reflected in the increased area under the ROC curves. These findings underscore the consistent and independent predictive role of the LDM/NDM ratio in the occurrence of vascular events.

Muscle mass, particularly in the lower extremity, decreases with age in both men and women. LDM area gradually declines in men with aging without an inflection point. In contrast, both LDM and NDM in women were maintained before the age of 55 years and thereafter both decreased with greater decrease in the NDM, consistent with a previous finding [7]. This finding suggests that muscle mass rapidly declines after menopause in women, indicating that hormonal changes are important in muscle maintenance. This may lead to increase in insulin resistance after menopause in women. The stability of the LDM/NDM ratio with age, in contrast to the decreasing trend in both LDM and NDM areas, suggests that this ratio may serve as a consistent indicator, irrespective of age-related changes in muscle mass. The LDM/NDM ratio, being a measure that considers the proportion of lipid-infiltrated muscle, captures alterations in muscle quality beyond mere muscle mass. The linear increasing trend in the incidence of vascular events across sex-specific quartiles of the LDM/NDM ratio was more prominent when compared to individual components like LDM area or other fat markers, such as BMI and VAT area.

The observed association between a higher LDM/NDM ratio at baseline and an increased incidence of vascular events over time implies a potential predictive value of this ratio in identifying individuals at greater risk for cardiovascular issues. This finding suggests that the LDM/NDM ratio may serve as an early indicator or marker for the development of vascular events. Although age, dyslipidemia, hypertension, and glycemic control are well-established components in the prevention of vascular events, it is important to note that, in our cohort, the LDM/NDM ratio remained statistically significant for both men and women, even after adjusting for all other risk factors. Furthermore, there was no significant correlation between the LDM/NDM ratio and established risk factors for vascular events, implying the independent role of the LDM/NDM ratio in the cardiovascular perspective. Therefore, our findings suggest that it could be a valuable addition to the existing risk factors for predicting future vascular events, especially CAD. Moreover, as mid-thigh LDM and NDM areas can be easily obtained from single cut CT scan without contrast dye, the use of LDM/NDM ratio may be advantageous in selecting persons who may be more vulnerable to vascular event. It is important to note, however, that the current analysis was based on the data of the Korean Atherosclerosis Study. Cardiac CT was recommended for participants in this cohort with diabetes mellitus or individuals with at least one cardiovascular risk factor. This explains the high diabetes prevalence (41.3%) in our cohort, a critical factor when interpreting the link between diabetes, cardiovascular risks, and muscle composition changes in our study.

Deleterious effects of ectopic fat accumulation in metabolically active tissues such as skeletal muscles have been well delineated in basic and clinical studies [19–25]. In the Framingham Heart Study, LDM at the paraspinal area was strongly related to metabolic risk factors [12]. A paradoxical inverse relationship of muscle attenuation and insulin resistance was detected in the Framingham Heart Study after adjusting for BMI and VAT, implicating that the apparent association of LDM to insulin resistance in the crude analysis may have only been an innocent bystander. In our study, the LDM/NDM ratio was an independent risk factor for vascular events while BMI and VAT were not. One difference between the two studies is the measurement site of muscle attenuation: paraspinous muscle vs. mid-thigh muscle. While low muscle radiodensity is presumed to reflect high levels of ectopic fat depots in muscles, stronger associations have been reported to triglyceride content have been reported in biopsied vastus lateralis muscles (*r* = −0.580) [26] as compared with muscle biopsies at the third lumbar vertebral level (*r* = −0.355) [9]. Thus, measuring muscle attenuation at the mid-thigh level and using its ratio to NDM area may represent ectopic fat accumulation more comprehensively as a constellation of metabolic abnormalities than other areas.

This study has some limitations. The use of a single precontrast cut at the umbilicus and mid-thigh level, aimed at minimizing radiation exposure, prevented the investigation of the association between liver fat—an important ectopic fat linked to glucose and lipid metabolism—and muscle fat in predicting vascular events. While measuring liver fat attenuation could enhance understanding, it would mean additional radiation exposure. In addition, when assessing adipose tissue, we opted to utilize the umbilicus as the reference point, aligning with the widely recognized waist circumference measurement site and consistent with prior research [27, 28]. Nonetheless, it is important to acknowledge that there is substantial support for considering L3 as the validated reference site for evaluating VAT.

In conclusion, our study demonstrated the significant role of the LDM/NDM ratio in predicting vascular events in both men and women. Although the contribution of the LDM/NDM ratio to this phenomenon may not be robust, its simplicity, reliability, and reproducibility in CT attenuation at the mid-thigh muscles suggest its potential as an adjunct marker for predicting future vascular events. Further research is warranted to explore the precise role of the LDM/NDM ratio in risk-prediction models and its utility in clinical practice.

### Electronic supplementary material

Below is the link to the electronic supplementary material.

Supplementary Material 1

## Data Availability

The data supporting this study’s findings are available from the corresponding author upon reasonable request.
