# Supplementary material for: Independent association of thigh muscle fat density with vascular events in Korean adults
Source: Cardiovasc Diabetol. 2024 Jan 28;23:44. doi: 10.1186/s12933-024-02138-w (PMC10823598; doi:10.1186/s12933-024-02138-w)
Supplement: Supplementary file 1 — Supplementary Material 1 [file 12933_2024_2138_MOESM1_ESM.docx]

**Figure S1.** Bland–Altman plot for intra-observer variability and inter-observer variability. Low density muscle (upper panel) and normal density muscle (lower panel).

**
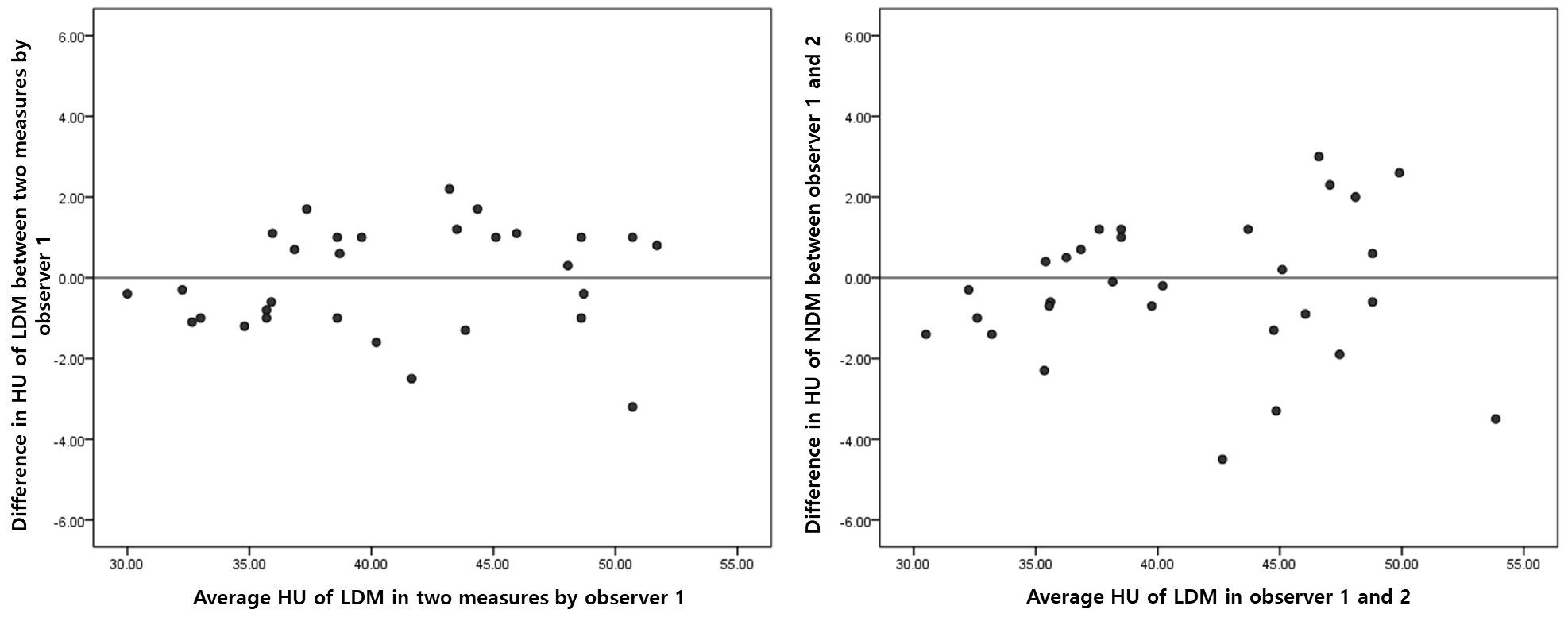
**

**
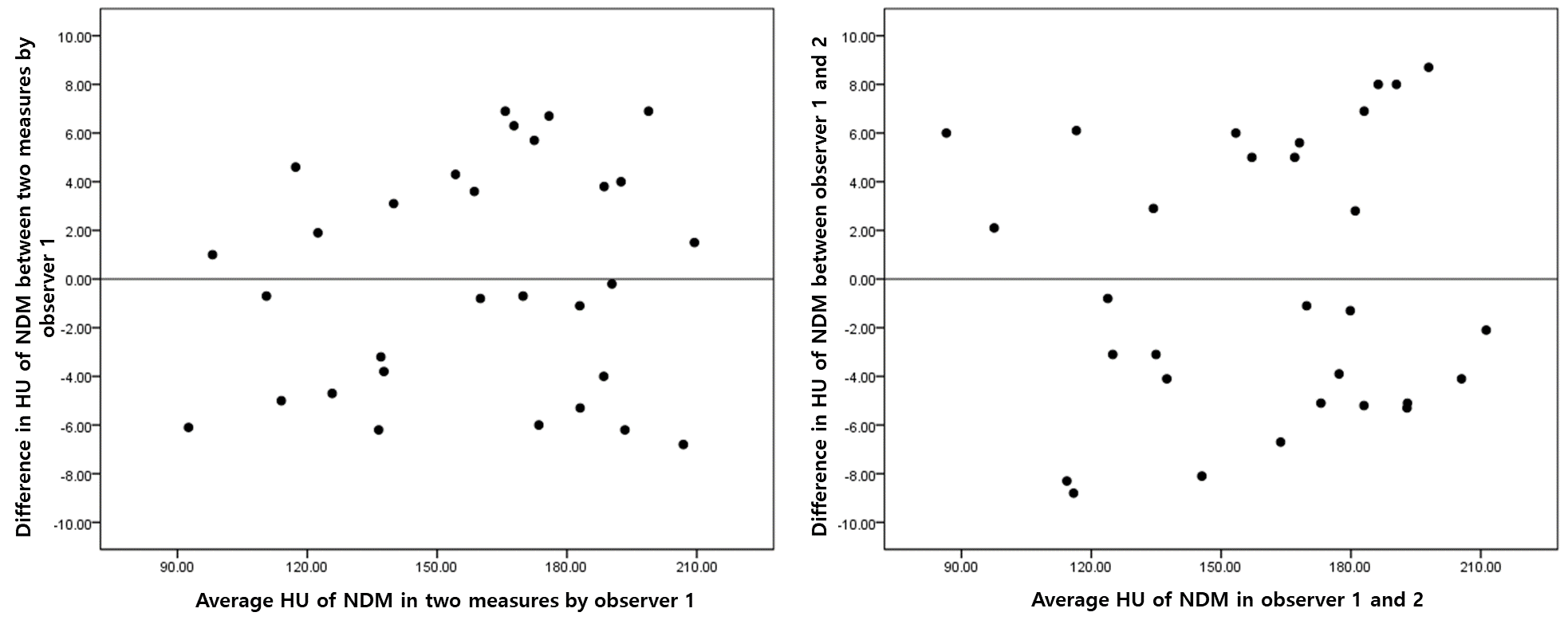
**

**Figure S2.** Boxplot for A. LDM area (cm^2^), B. NDM area (cm^2^), and C. LDM/NDM ratio (%) of men and women according to age groups. The box represents the interquartile range (IQR), upper whiskers extend to the maximum value of the data within 1.5 times the IQR over the 75^th^ percentile, and the lower whiskers is the minimum value of the data within 1.5 times the interquartile range under the 25^th^ percentile.


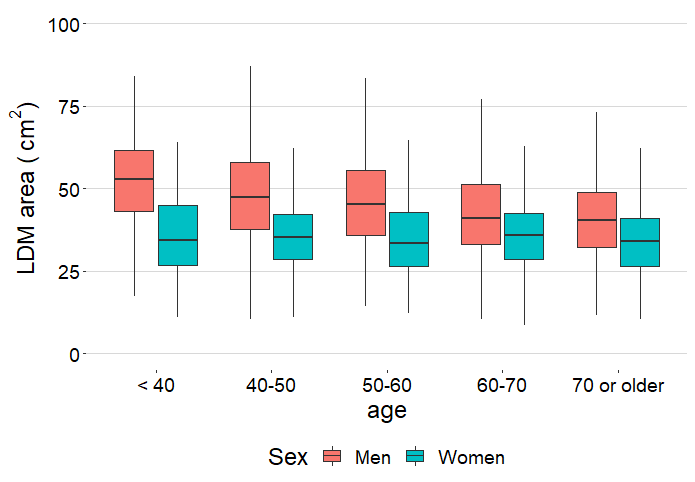

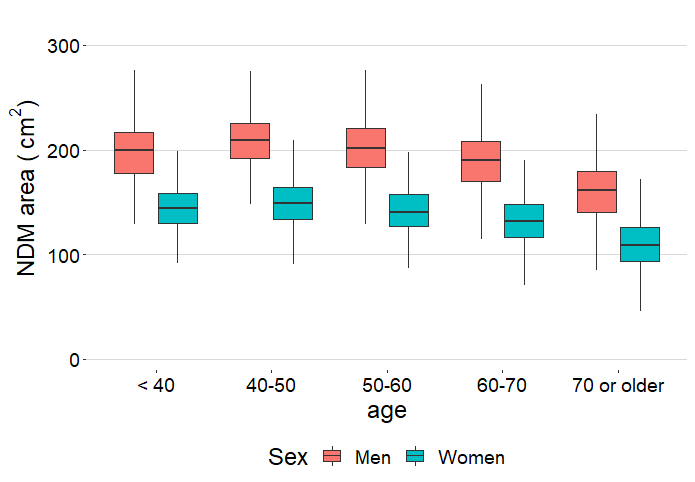

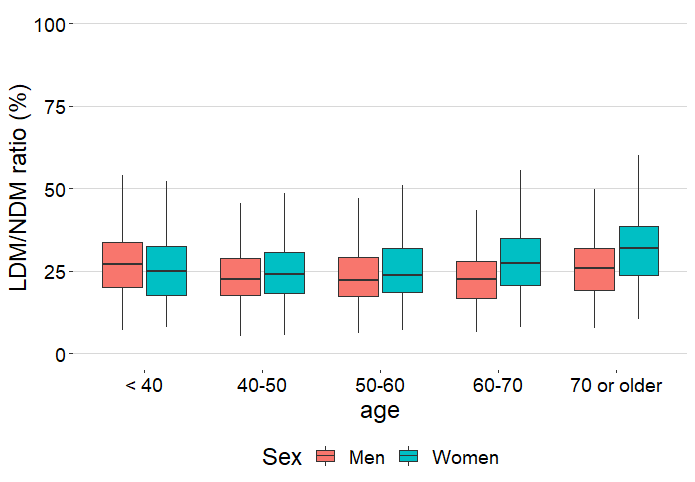

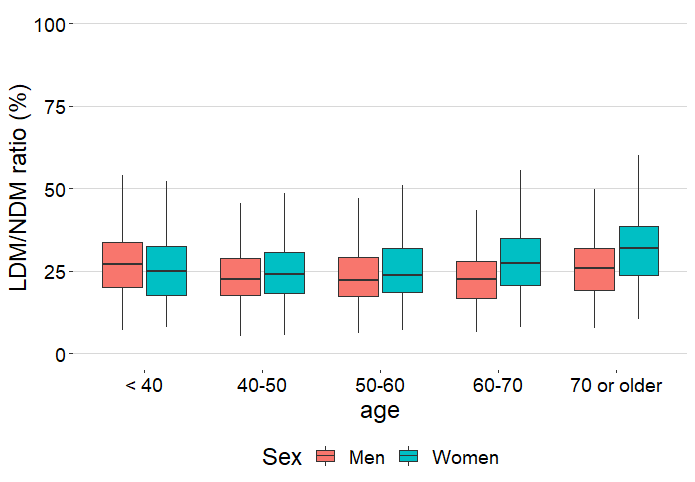


**A**

**B**

**C**

**Figure S3.** Incidence of vascular events (%) according to sex-specific quartiles of LDM/NDM ratio, LDM area, VAT, and body mass index (BMI). Red and green bars represent men and women, respectively. *P* for trend is listed above the bars.

| **Male** | **LDM/NDM ratio (%)** | **LDM area (cm^2^)** | **VAT (cm^2^)** | **BMI (kg/m^2^)** |
| --- | --- | --- | --- | --- |
| **Q1** | Min-18.0 | Min-35.1 | Min-101.3 | Min-23.5 |
| **Q2** | 18.0-23.5 | 35.1-45.2 | 101.3-140.0 | 23.5-25.4 |
| **Q3** | 23.5-30.5 | 45.2-55.2 | 140.1-179.0 | 25.4-27.6 |
| **Q4** | 30.5-Max | 55.2-Max | 179.0-Max | 27.6-Max |
|  |  |  |  |  |
| **Female** | **LDM/NDM ratio (%)** | **LDM area (cm^2^)** | **VAT (cm^2^)** | **BMI (kg/m^2^)** |
| **Q1** | Min-19.7 | Min-27.1 | Min-75.4 | Min-22.5 |
| **Q2** | 19.7-26.1 | 27.1-34.5 | 75.5-110.5 | 23.5-25.4 |
| **Q3** | 26.1-34.8 | 34.6-42.4 | 110.5-146.3 | 25.4-27.6 |
| **Q4** | 34.8-Max | 42.5-Max | 146.5-Max | 27.6-Max |

**Figure S4.** Correlation plot. Correlograms for conventional cardiometabolic risk factors with LDM area and LDM/NDM ratio in men (A) and women (B). Positive association shaded in blue, negative association shaded in red. Numbers inside the box indicate the Pearson’s correlation coefficient, ρ. *P* values for the correlation coefficient for the risk factors and the LDM/NDM ratio (%) are listed in the table. VAT, visceral adipose tissue; C, cholesterol; LDM, low density muscle.

**
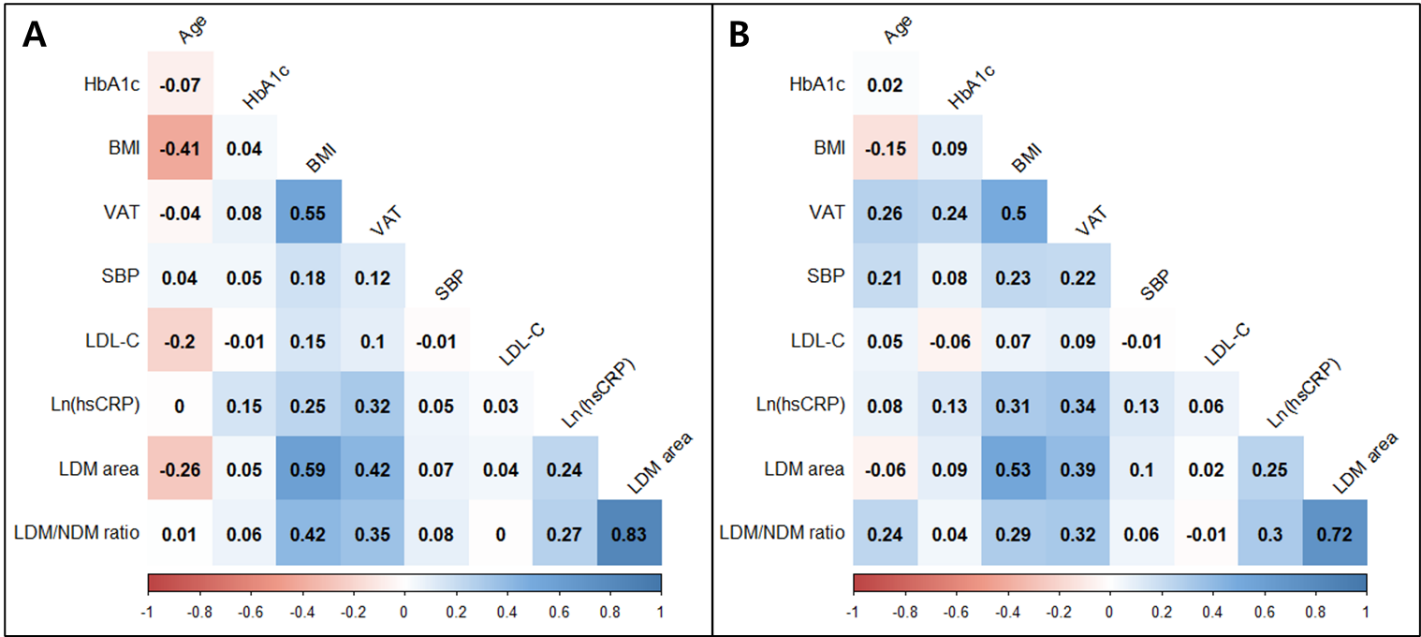
**

| **LDM/NDM ratio (%)** | | | | | | | | | |
| --- | --- | --- | --- | --- | --- | --- | --- | --- | --- |
| **Men** | **Age** | **HbA1c** | **BMI** | **VAT** | **SBP** | **LDL-C** | **Ln(hsCRP)** | **LDM area** |  |
| **ρ** | 0.008 | 0.064 | 0.416 | 0.350 | 0.083 | -0.005 | 0.267 | 0.832 |  |
| ***P*** | 0.354 | 0.592 | 0.101 | 0.232 | 0.628 | 0.534 | 0.578 | <0.001 |  |
| **Women** |  |  |  |  |  |  |  |  |  |
| **ρ** | 0.237 | 0.041 | 0.291 | 0.315 | 0.056 | -0.011 | 0.303 | 0.719 |  |
| ***P*** | 0.924 | 0.469 | 0.449 | 0.592 | 0.418 | 0.313 | 0.587 | 0.005 |  |

**Figure S5.** Receiver operating characteristics curves for vascular events in A. men and B. women.

**
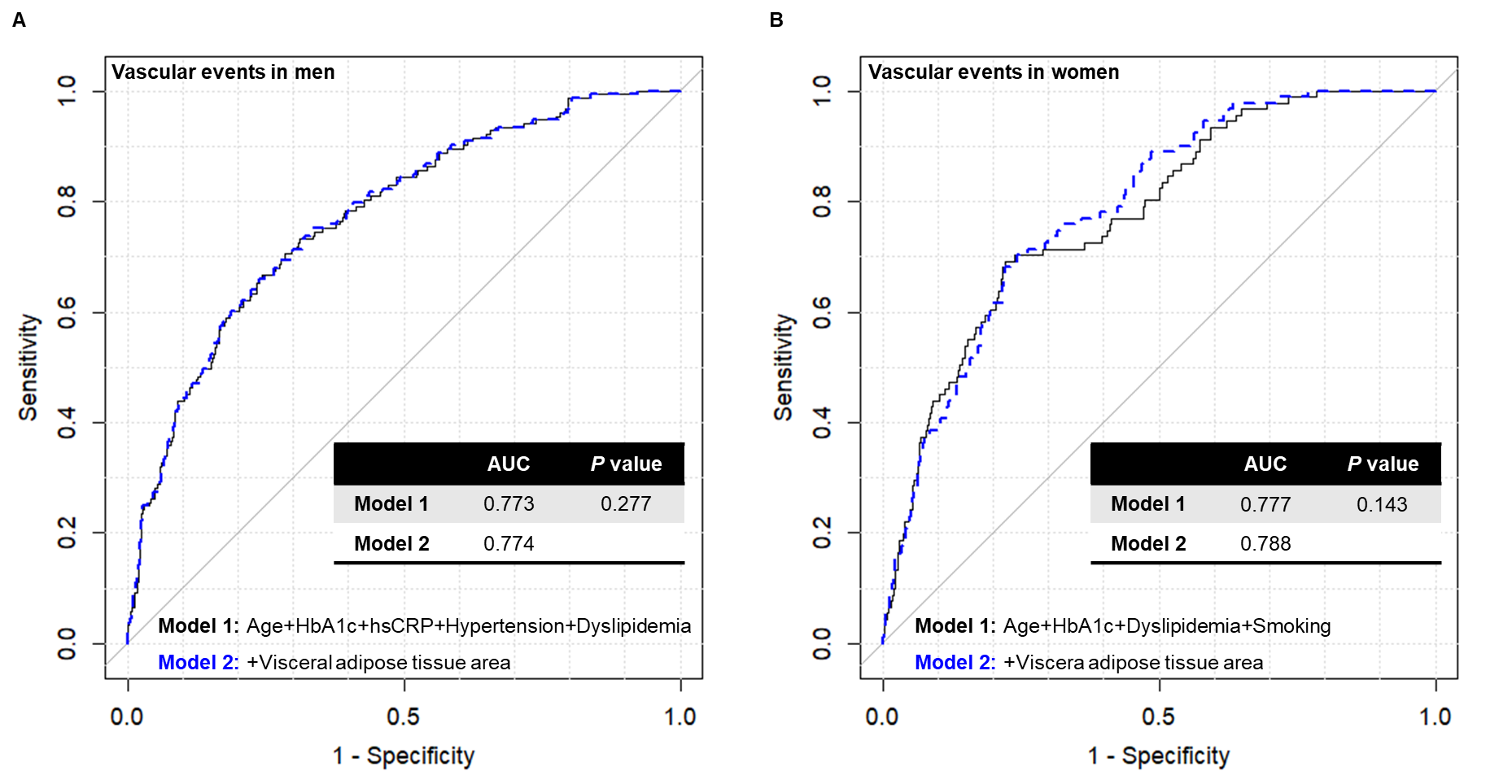
**

**Figure S6.** Receiver operating characteristics curves for coronary artery disease in A. men and B. women.

**
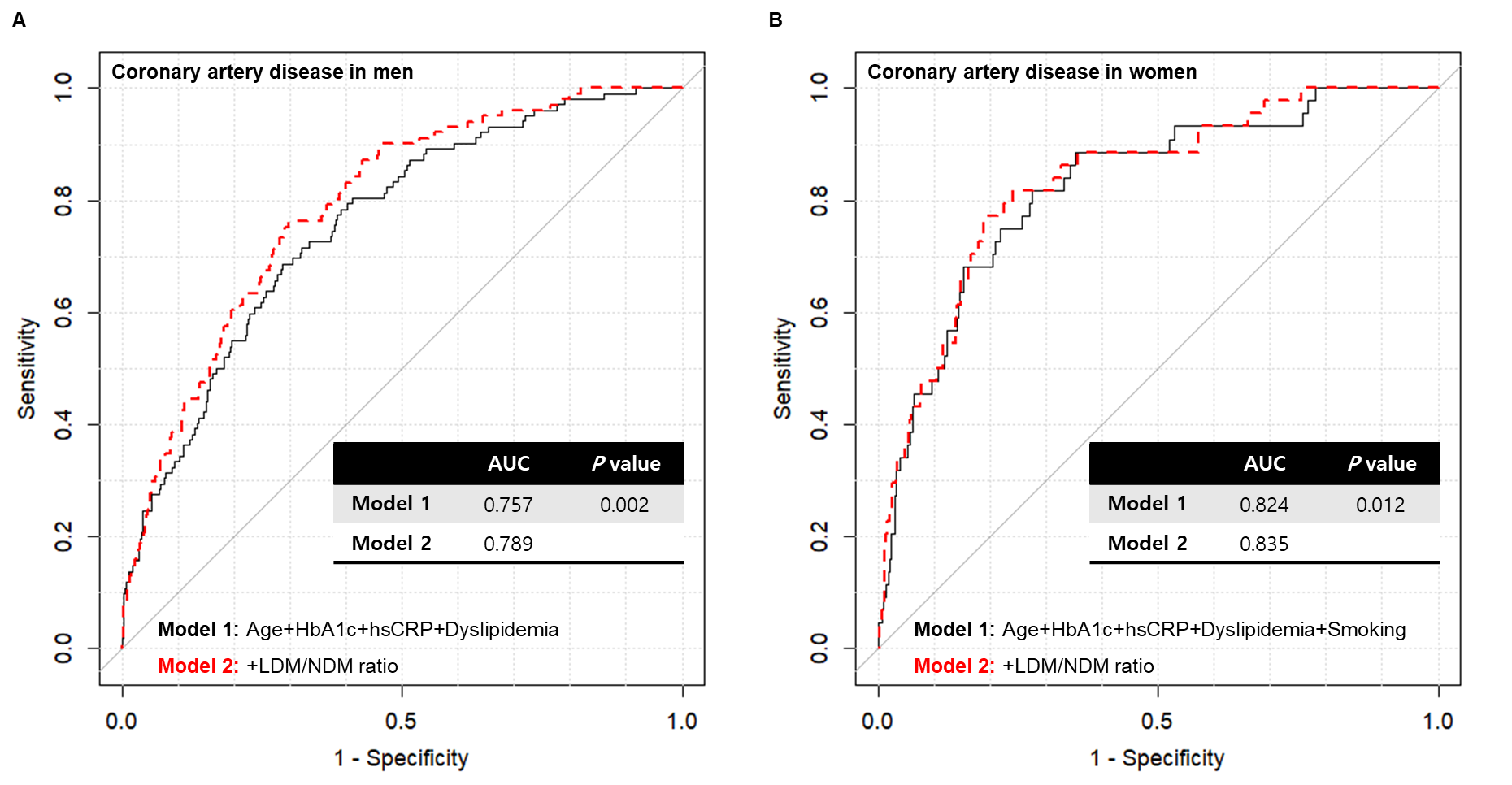
**

**Figure S7.** Receiver operating characteristics curves for cerebrovascular disease in A. men and B. women.


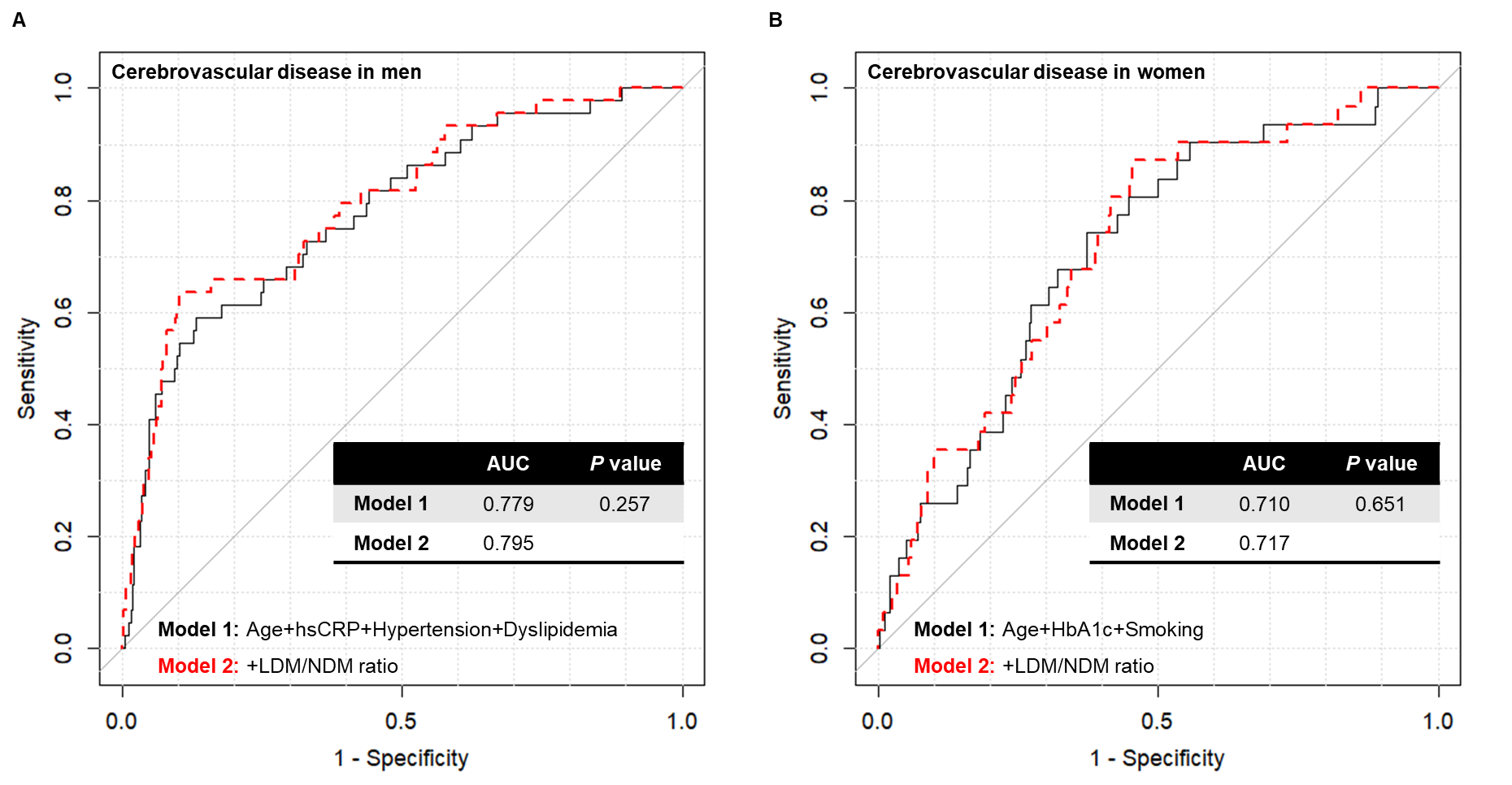


**Figure S8.** Receiver operating characteristics curves for peripheral artery disease in A. men and B. women.


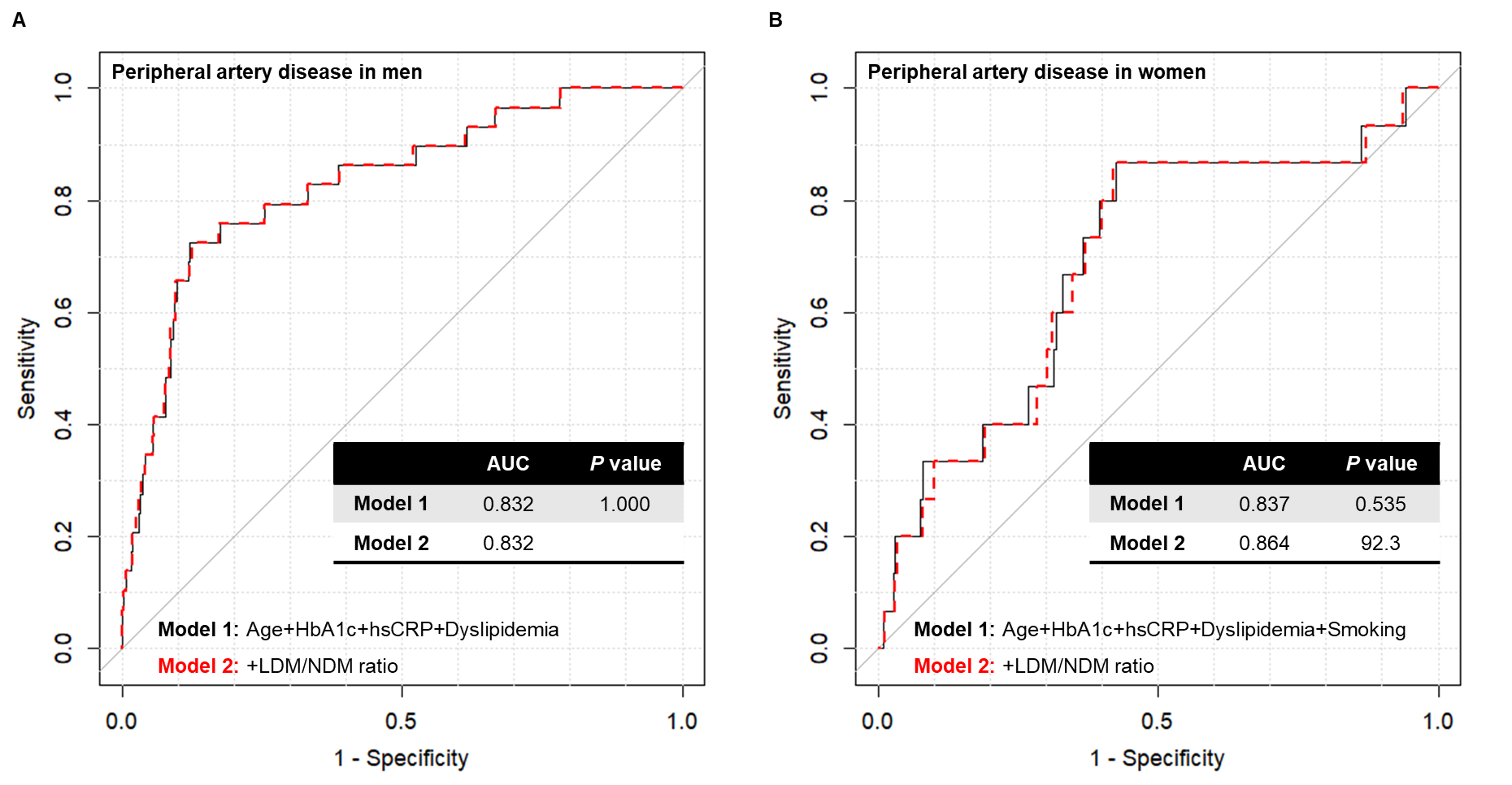


| **Table S1.** Intra-observer and inter-observer variability in measurement of HU for low density muscle (LDM) and normal density muscle (NDM) among 30 individuals of study population. | | | | | |
| --- | --- | --- | --- | --- | --- |
|  | First measure by observer 1 (HU) | Second measure by observer 1 (HU) | First measure – Second measure (HU) | *P* | ICC |
| LDM kg/m^2^ | 41.00 ± 6.36 | 41.03 ± 6.18 | –0.033 ± 1.31 | 0.890 | 0.989 |
| NDM | 158.81 ± 33.67 | 158.82 ± 32.96 | –0.017 ± 4.65 | 0.984 | 0.995 |
|  | Measure by observer 1 (HU) | Measure by observer 2 (HU) | Observer 1–Observer 2 (HU) |  |  |
| LDM | 41.00 ± 6.36 | 41.26 ± 6.21 | –0.260 ± 1.77 | 0.428 | 0.980 |
| NDM | 158.81 ± 33.67 | 158.91 ± 33.3 | –0.103 ± 5.58 | 0.920 | 0.993 |
| Data are expressed as the mean ± SD. P values by paired t test between first measure and second measure. ICC: Intraclass Correlation Coefficient. | | | | | |

| **Table S2. Baseline characteristics of the participants with or without vascular events at baseline** | | | | | | |
| --- | --- | --- | --- | --- | --- | --- |
|  | Men (N = 1,971) | | | Women (N = 1,030) | | |
|  | Previous VE | Included | *P* | Previous VE | Included | *P* |
|  | N = 84 | N = 1,887 |  | N = 56 | N = 1,722 |  |
| Age (years) | 64.4 ± 11.5 | 56.4 ± 15.9 | <0.001 | 71.8 ± 9.2 | 59.2 ± 15.7 | <0.001 |
| BMI (kg/m^2^) | 25.4 ± 3.5 | 25.7 ± 3.9 | 0.510 | 25.2 ± 4 | 25.3 ± 4.1 | 0.949 |
| SBP (mmHg) | 127 ± 16 | 130 ± 16 | 0.118 | 130 ± 16 | 129 ± 16 | 0.556 |
| DBP (mmHg) | 75 ± 11 | 79 ± 11 | 0.003 | 73 ± 9 | 76 ± 11 | 0.045 |
| HbA1c (%) | 7.2 ± 1.8 | 6.7 ± 1.4 | 0.022 | 7.0 ± 1.1 | 6.5 ± 1.3 | 0.014 |
| Fasting glucose (mg/dL) | 131 ± 44 | 123 ± 39 | 0.117 | 118 ± 31 | 114 ± 35 | 0.443 |
| Total cholesterol (mg/dL) | 167 ± 36 | 192 ± 37 | <0.001 | 176 ± 37 | 200 ± 38 | <0.001 |
| Triglyceride (mg/dL) | 143 ± 89 | 157 ± 111 | 0.437 | 131 ± 83 | 137 ± 83 | 0.830 |
| HDL-cholesterol (mg/dL) | 46.4 ± 13.8 | 50 ± 12 | 0.023 | 51 ± 14 | 56 ± 14 | 0.015 |
| LDL-cholesterol (mg/dL) | 87 ± 22 | 107 ± 30 | <0.001 | 84 ± 26 | 107 ± 29 | <0.001 |
| Total protein (g/dL) | 7.1 ± 0.5 | 7.2 ± 0.5 | 0.359 | 7.1 ± 0.6 | 7.2 ± 0.4 | 0.348 |
| AST (IU/L) | 27 ± 19 | 29 ± 73 | 0.817 | 23 ± 8 | 24 ± 13 | 0.414 |
| ALT (IU/L) | 31 ± 25 | 36 ± 72 | 0.609 | 22 ± 12 | 24 ± 20 | 0.324 |
| Creatinine (mg/dL) | 1.02 ± 0.51 | 1.01 ± 0.40 | 0.932 | 0.91 ± 0.53 | 0.79 ± 0.40 | 0.090 |
| eGFR (mL/min/1.73 m^2^) | 71.5 ± 13.5 | 77.0 ± 13.6 | 0.009 | 61.0 ± 18.2 | 72.2 ± 14.1 | <0.001 |
| Urinary microalbumin/Cr | 146 ± 630 | 52 ± 266 | 0.001 | 109 ± 332 | 40 ± 170 | 0.017 |
| hsCRP (mg/L) | 1.11 ± 2.82 | 0.49 ± 1.63 | 0.024 | 0.76 ± 1.42 | 0.30 ± 0.93 | 0.001 |
| *Comorbidity* |  |  |  |  |  |  |
| Hypertension, n (%) | 51 (60.7) | 651 (34.5) | <0.001 | 32 (57.1) | 576 (33.5) | <0.001 |
| Dyslipidemia, n (%) | 50 (59.5) | 827 (43.8) | 0.005 | 34 (60.7) | 742 (43.1) | 0.009 |
| Diabetes mellitus, n (%) | 50 (68.5) | 877 (50.4) | 0.002 | 31 (63.3) | 615 (38.9) | <0.001 |
| **Abdomen** |  |  |  |  |  |  |
| TAT (cm^2^) | 290.3 ± 96.5 | 294.6 ± 118.8 | 0.744 | 340.3 ± 121.7 | 330.1 ± 118 | 0.525 |
| VAT (cm^2^) | 142.5 ± 53.5 | 142.0 ± 60.1 | 0.944 | 132.6 ± 55.7 | 113.8 ± 52.1 | 0.008 |
| SAT (cm^2^) | 147.8 ± 58.9 | 152.5 ± 77.5 | 0.577 | 207.7 ± 90.9 | 216.3 ± 85.0 | 0.456 |
| **Mid-thigh area** |  |  |  |  |  |  |
| Fat area (cm^2^) | 91.5 ± 47.5 | 94.1 ± 50.0 | 0.636 | 128.8 ± 43.5 | 153.4 ± 65.0 | <0.001 |
| Muscle area (cm^2^) | 224.6 ± 38.0 | 237.9 ± 38.4 | 0.002 | 152.5 ± 31.5 | 168.2 ± 31.2 | <0.001 |
| LDM (0–30 HU) (cm^2^) | 46.0 ± 13.2 | 45.8 ± 14.7 | 0.910 | 39.7 ± 11.1 | 35.5 ± 11.8 | 0.010 |
| NDM (31–100 HU) (cm^2^) | 178.7 ± 34.3 | 192.2 ± 35.2 | 0.001 | 112.8 ± 29.9 | 132.7 ± 28.7 | <0.001 |
| LDM/NDM ratio (%) | 26.6 ± 9.4 | 24.6 ± 9.2 | 0.056 | 38.3 ± 17.2 | 28.4 ± 12.9 | <0.001 |
| VE, vascular event; LDM, low density muscle; NDM, normal density muscle. | | | | | | |

| **Table S3. Risk factors in the development of coronary artery disease** | | | | | | | | |
| --- | --- | --- | --- | --- | --- | --- | --- | --- |
|  | Men (N = 1,887) | | | | Women (N = 1,722) | | | |
|  | Univariable | | Multivariable | | Univariable | | Multivariable | |
|  | Unadjusted | *P* | Adjusted HR | *P* | Unadjusted | *P* | Adjusted HR | *P* |
|  | HR (95% CI) |  | (95% CI) |  | HR (95% CI) |  | (95% CI) |  |
| LDM/NDM ratio (%) | 1.07 (1.05–1.09) | <0.001 | 1.05 (1.03–1.07) | <0.001 | 1.04 (1.03–1.05) | <0.001 | 1.02 (1.00–1.04) | 0.032 |
| Age (years) | 1.04 (1.03–1.06) | <0.001 | 1.06 (1.04–1.08) | <0.001 | 1.08 (1.05–1.10) | <0.001 | 1.07 (1.03–1.12) | <0.001 |
| HbA1c (%) | 1.32 (1.20–1.45) | <0.001 | 1.27 (1.10–1.48) | 0.001 | 1.29 (1.09–1.51) | 0.002 | 1.25 (1.01–1.55) | 0.040 |
| eGFR (mL/min/1.73m²) | 0.99 (0.98–1.00) | 0.152 | 1.00 (0.99–1.02) | 0.798 | 0.98 (0.96–1.00) | 0.075 | 1.01 (0.98–1.03) | 0.649 |
| hsCRP (mg/L)* | 1.29 (1.18–1.41) | <0.001 | 1.13 (1.02–1.26) | 0.017 | 1.51 (1.29–1.76) | <0.001 | 1.33 (1.11–1.59) | 0.002 |
| Visceral adipose tissue (cm^2^) | 1.00 (1.00–1.00) | <0.001 | 1.00 (1.00–1.00) | 0.846 | 1.00 (1.00–1.00) | <0.001 | 1.00 (0.99–1.01) | 0.710 |
| Hypertension, yes | 3.02 (2.16–4.22) | <0.001 | 1.53 (0.98–2.38) | 0.059 | 3.79 (2.05–7.00) | <0.001 | 1.78 (0.89–3.58) | 0.104 |
| Dyslipidemia, yes | 3.32 (2.29–4.79) | <0.001 | 2.98 (1.83–4.85) | <0.001 | 3.86 (1.88–7.92) | <0.001 | 4.92 (1.83–13.25) | 0.002 |
| Smoking |  | 0.095 |  | 0.069 |  | 0.014 |  | <0.001 |
| Never smoker | 1 (Ref) |  | 1 (Ref) |  | 1 (Ref) |  | 1 (Ref) |  |
| Ex-smoker | 0.98 (0.66–1.46) | 0.919 | 1.34 (0.83–2.16) | 0.228 | 3.03 (0.94–9.75) | 0.063 | 9.53 (2.69–33.73) | <0.001 |
| Current smoker | 1.51 (1.00–2.27) | 0.050 | 1.67 (0.94–2.98) | 0.083 | 3.70 (0.89–15.32) | 0.071 | 7.30 (0.92–57.95) | 0.060 |
| CI, confidence interval; HR, hazard ratio; LDM, low density muscle; NDM, normal density muscle; hsCRP, high sensitivity C-reactive protein; eGFR, estimated glomerular filtration rate. *Log transformed for analysis | | | | | | | | |

| **Table S4. Risk factors in the development of cerebrovascular disease** | | | | | | | | |
| --- | --- | --- | --- | --- | --- | --- | --- | --- |
|  | Men (N = 1,887) | | | | Women (N = 1,722) | | | |
|  | Univariable | | Multivariable | | Univariable | | Multivariable | |
|  | Unadjusted | *P* | Adjusted HR | *P* | Unadjusted | *P* | Adjusted HR | *P* |
|  | HR (95% CI) |  | (95% CI) |  | HR (95% CI) |  | (95% CI) |  |
| LDM/NDM ratio (%) | 1.07 (1.04–1.10) | <0.001 | 1.06 (1.02–1.09) | 0.001 | 1.03 (1.02–1.05) | <0.001 | 1.02 (0.99–1.04) | 0.138 |
| Age (years) | 1.07 (1.05–1.10) | <0.001 | 1.08 (1.05–1.11) | <0.001 | 1.09 (1.05–1.13) | <0.001 | 1.10 (1.05–1.15) | <0.001 |
| HbA1c (%) | 1.11 (0.91–1.36) | 0.297 | 1.04 (0.78–1.38) | 0.789 | 1.21 (0.98–1.49) | 0.077 | 1.33 (1.04–1.71) | 0.023 |
| eGFR (mL/min/1.73m²) | 0.98 (0.95–1.00) | 0.026 | 1.01 (0.98–1.03) | 0.600 | 0.98 (0.95–1.01) | 0.136 | 1.00 (0.97–1.03) | 0.908 |
| hsCRP (mg/L)* | 1.27 (1.10–1.47) | <0.001 | 1.22 (1.04–1.43) | 0.012 | 1.21 (1.01–1.44) | 0.036 | 1.01 (0.81–1.25) | 0.942 |
| Visceral adipose tissue (cm^2^) | 1.00 (1.00–1.00) | 0.234 | 0.99 (0.99–1.00) | 0.112 | 1.00 (1.00–1.00) | 0.006 | 1.00 (0.99–1.01) | 0.496 |
| Hypertension, yes | 3.39 (1.94–5.91) | <0.001 | 2.48 (1.22–5.07) | 0.012 | 1.81 (0.93–3.49) | 0.079 | 0.91 (0.43–1.93) | 0.797 |
| Dyslipidemia, yes | 2.11 (1.22–3.65) | 0.008 | 2.66 (1.31–5.39) | 0.007 | 1.19 (0.61–2.31) | 0.612 | 1.07 (0.47–2.40) | 0.877 |
| Smoking |  | 0.685 |  | 0.612 |  | 0.011 |  | 0.002 |
| Never smoker | 1 (Ref) |  | 1 (Ref) |  | 1 (Ref) |  | 1 (Ref) |  |
| Ex-smoker | 1.18 (0.65–2.15) | 0.593 | 1.22 (0.59–2.53) | 0.598 | 2.99 (0.72–12.51) | 0.133 | 7.17 (1.50–34.28) | 0.014 |
| Current smoker | 0.73 (0.30–1.73) | 0.470 | 1.22 (0.43–3.46) | 0.714 | 4.84 (1.15–20.34) | 0.031 | 10.08 (1.17–86.64) | 0.035 |
| CI, confidence interval; HR, hazard ratio; LDM, low density muscle; NDM, normal density muscle; hsCRP, high sensitivity C-reactive protein; eGFR, estimated glomerular filtration rate. *Log transformed for analysis | | | | | | | | |

| **Table S5. Risk factors in the development of peripheral artery disease** | | | | | | | | |
| --- | --- | --- | --- | --- | --- | --- | --- | --- |
|  | Men (N = 1,887) | | | | Women (N = 1,722) | | | |
|  | Univariable | | Multivariable | | Univariable | | Multivariable | |
|  | Unadjusted | *P* | Adjusted HR | *P* | Unadjusted | *P* | Adjusted HR | *P* |
|  | HR (95% CI) |  | (95% CI) |  | HR (95% CI) |  | (95% CI) |  |
| LDM/NDM ratio (%) | 1.02 (0.99–1.06) | 0.193 | 1.02 (0.98–1.06) | 0.298 | 1.03 (1.01–1.05) | 0.001 | 1.02 (0.99–1.05) | 0.123 |
| Age (years) | 1.03 (1.01–1.06) | 0.015 | 1.08 (1.04–1.12) | <0.001 | 1.03 (0.99–1.06) | 0.136 | 1.06 (1.00–1.11) | 0.047 |
| HbA1c (%) | 1.60 (1.40–1.83) | <0.001 | 1.71 (1.36–2.14) | <0.001 | 1.60 (1.36–1.87) | <0.001 | 1.59 (1.26–2.00) | <0.001 |
| eGFR (mL/min/1.73m²) | 1.01 (0.99–1.03) | 0.553 | 1.01 (0.99–1.03) | 0.450 | 0.99 (0.96–1.02) | 0.326 | 1.00 (0.97–1.03) | 0.989 |
| hsCRP (mg/L)* | 1.37 (1.15–1.63) | <0.001 | 1.23 (1.02–1.48) | 0.031 | 1.30 (1.04–1.62) | 0.019 | 1.17 (0.91–1.50) | 0.224 |
| Visceral adipose tissue (cm^2^) | 1.00 (1.00–1.00) | 0.887 | 1.00 (0.99–1.00) | 0.528 | 1.00 (1.00–1.00) | 0.051 | 1.00 (0.99–1.01) | 0.883 |
| Hypertension, yes | 1.59 (0.84–3.01) | 0.152 | 0.92 (0.41–2.07) | 0.840 | 1.89 (0.87–4.13) | 0.108 | 1.18 (0.47–2.95) | 0.718 |
| Dyslipidemia, yes | 4.13 (1.89–9.02) | <0.001 | 2.91 (1.18–7.20) | 0.021 | 4.45 (1.53–12.92) | 0.006 | 3.42 (0.97–12.14) | 0.057 |
| Smoking |  | 0.020 |  | 0.504 |  | 0.024 |  | 0.459 |
| Never smoker | 1 (Ref) |  | 1 (Ref) |  | 1 (Ref) |  | 1 (Ref) |  |
| Ex-smoker | 1.19 (0.53–2.67) | 0.675 | 0.83 (0.31–2.22) | 0.706 | 4.27 (1.00–18.14) | 0.049 | 3.11 (0.48–20.17) | 0.235 |
| Current smoker | 2.56 (1.22–5.37) | 0.013 | 1.52 (0.57–4.03) | 0.404 | 4.43 (0.60–32.83) | 0.145 | N/A | 0.983 |
| CI, confidence interval; HR, hazard ratio; LDM, low density muscle; NDM, normal density muscle; hsCRP, high sensitivity C-reactive protein; eGFR, estimated glomerular filtration rate; N/A, not applicable. *Log transformed for analysis | | | | | | | | |
